# Genotypic Analysis of Piroplasms and Associated Pathogens from Ticks Infesting Cattle in Korea

**DOI:** 10.3390/microorganisms8050728

**Published:** 2020-05-13

**Authors:** Min-Goo Seo, Oh-Deog Kwon, Dongmi Kwak

**Affiliations:** 1 Veterinary Drugs and Biologics Division, Animal and Plant Quarantine Agency, 177 Hyeoksin 8-ro, Gimcheon, Gyeongbuk 39660, Korea; koreasmg@korea.kr; 2 Department of Veterinary Medicine, College of Veterinary Medicine, Kyungpook National University, 80 Daehak-ro, Buk-gu, Daegu 41566, Korea; odkwon@knu.ac.kr; 3 Cardiovascular Research Institute, Kyungpook National University, 680 Gukchaebosang-ro, Jung-gu, Daegu 41944, Korea

**Keywords:** tick-borne pathogens, phylogeny, Theileria, Anaplasma, Ehrlichia, Rickettsia, Haemaphysalis longicornis

## Abstract

Tick-borne pathogens (TBPs) impose an important limitation to livestock production worldwide, especially in subtropical and tropical areas. Earlier studies in Korea have examined TBPs residing in ticks and animals; however, information on multiple TBPs in ticks infesting cattle is lacking. This study assessed the prevalence of TBPs in ticks parasitizing cattle. A total of 576 ticks, including 340 adults and 236 nymphs, were collected from cattle in Korea between 2014 and 2018. All ticks collected were identified as *Haemaphysalis longicornis* based on their morphological and molecular characteristics. Among piroplasms and other tick-associated pathogens, seven TBP genes, namely *Theileria orientalis* (5.0%), *Anaplasma bovis* (2.3%), *Anaplasma capra* (4.7%), *Anaplasma phagocytophilum*-like *Anaplasma* spp. (APL) clades A (1.9%) and B (0.5%), *Ehrlichia canis* (1.6%), and *Candidatus* Rickettsia longicornii (17.5%), were detected. *Bartonella* spp. and severe fever with thrombocytopenia syndrome virus were not found. To our knowledge, this is the first study to report the presence of the pathogens *T. orientalis* major piroplasm surface protein genotypes 3 and 7, *A. capra*, and APL in ticks from Korea. Cattle ticks may be maintenance hosts for many TBPs, and veterinary and medical clinicians should be aware of their high probability of infection and clinical complexity in humans.

## 1. Introduction 

Many emergent tick-borne pathogens (TBPs) were circulating in animals and ticks long before their identification as causes of clinical diseases [1]. The global hazard of TBPs is increasing and fostering public health concerns, as novel pathogens have been continuously recognized during the past two decades [2]. The capability of ticks to attach to humans and transmit pathogens is affected by several factors, including human activities, geographical and climatic conditions, and tick abundance, biological stage, burden, and attachment duration [3].

TBPs impose an important limitation to livestock production across the world, especially in subtropical and tropical areas [4]. Much of the world’s cattle population is influenced by ticks and TBPs, and the damage caused by them is extremely high [5]. Due to the significance of hard ticks (Acari: Ixodidae) in veterinary medicine and the financial cost of their control, the transmission of tick-borne diseases remains an issue for the cattle industry in subtropical and tropical regions, and it is a primary concern for numerous countries in these areas [6].

Understanding the ecology of local tick species parasitizing animals and identifying the TBPs they carry are of paramount public health significance. Previous studies have examined the presence of TBPs in Korea by studying ticks and cattle using molecular methods [7,8,9,10,11,12,13,14]. The current study measured the prevalence of TBPs, including piroplasms (genera *Babesia* and *Theileria*)*,* rickettsiae (genera *Anaplasma*, *Ehrlichia*, and *Rickettsia*), *Bartonella* spp., and severe fever with thrombocytopenia syndrome virus (SFTSV), in ticks collected from cattle in Korea.

## 2. Materials and Methods

### 2.1. Ethical Approval

Approval from Kyungpook National University’s Institutional Animal Care and Use Committee was not required for the present study conducted from 2014 to 2018. With oral permission from cattle farm owners, veterinarians from local veterinary institutes collected tick samples when the animals underwent treatment, surveillance, monitoring, or regular check-ups. Tick removal did not cause pain or physical injury to any animals in the study.

### 2.2. Tick Collection and Species Identification

In total, 576 ticks, including 236 nymphs and 340 adults, were collected from cattle in the northern (81 from Gyeonggi and 82 from Gangwon), central (35 from Chungbuk, 45 from Chungnam, and 77 from Gyeongbuk), and southern (75 from Jeonbuk, 88 from Jeonnam, and 93 from Gyeongnam) areas of Korea from 2014 to 2018. Four to fifteen ticks for each animal were collected by a simple random sampling method from 149 cattle (Hanwoo, native Korean cattle, *Bos taurus coreanae*) and then stored in tubes containing 70% ethanol. The collected ticks were primarily identified by their morphological features [15], with an additional classification by the molecular methods described below.

### 2.3. Molecular Detection of Ticks and TBPs

Genomic DNA was extracted from the ticks using a commercial DNeasy Blood & Tissue Kit (Qiagen, Melbourne, Australia), according to the manufacturer’s instructions. The AccuPower HotStart PCR Premix Kit (Bioneer, Daejeon, Korea) was employed for the PCR amplification. Molecular identification of tick species was performed by amplifying the sequence of the mitochondrial cytochrome c oxidase subunit I (*COI*) gene using specific primers as described previously [16].

The ticks were then screened for several TBPs using primer sets specific to each pathogen. To detect piroplasm 18S rRNA, samples were first examined for infection by piroplasms by PCR using a commercial AccuPower *Babesia* and *Theileria* PCR Kit (Bioneer). Positive samples were then re-amplified by PCR using primers designed from the common sequence of the 18S rRNA gene of numerous piroplasm species [17], and major piroplasm surface protein (MPSP) genes of *Theileria* species were amplified by PCR [18]. Infection with rickettsiae was primarily tested by PCR via a commercial AccuPower Rickettsiales 3-Plex PCR Kit (Bioneer) for the detection of rickettsiae 16S rRNA. Additionally, positive samples were submitted to amplification for species identification. Positive samples of *Anaplasma* spp. were confirmed by amplifying 16S rRNA fragments using nested PCR (nPCR) [12,13], while positive samples of *Rickettsia* spp. were confirmed by PCR, which targeted the citrate synthase gene (*gltA*) [19]. nPCR was employed to amplify the internal transcribed spacer region sequence of *Bartonella* spp. [20] and the S segment of SFTSV [21].

All primers and amplification conditions used for detecting TBPs in ticks from cattle in the current study are described in Supplementary Table S1.

### 2.4. DNA Cloning, Nucleotide Sequencing, and Phylogenetic Analysis

For positive PCR products, DNA cloning was performed using pGEM-T Easy vectors (Promega, Madison, WI, USA) and *Escherichia coli* DH5α-competent cells (Thermo Fisher Scientific, Wilmington, DE, USA). Following cloning, nucleotide sequencing was performed with the multiple sequence alignment program CLUSTAL Omega (v. 1.2.1) [22]. Sequence alignment results were modified by BioEdit (v. 7.2.5) [23]. Phylogenetic analysis was conducted using MEGA (v. 6.0) [24], according to the maximum likelihood method with the Kimura two-parameter distance model. A similarity matrix was used to analyze the aligned sequences. The trees’ stability was assessed by a bootstrap analysis using 1000 replicates.

### 2.5. Statistical Analysis

The two-sided Fisher’s exact test was performed to analyze significant differences between pathogens for each tick stage, and a value of *p* < 0.05 was considered to indicate statistical significance. GraphPad Prism (v. 8.0; GraphPad Software Inc., La Jolla, CA, USA) was employed for the statistical analyses.

## 3. Results

### 3.1. Identification of Ticks

**Figure 1:**
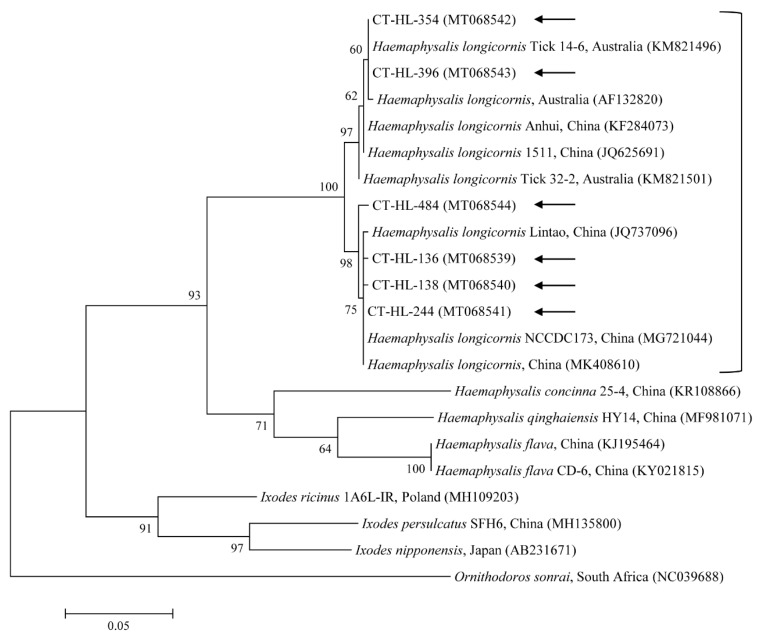
Molecular identification of ticks according to phylogenetic analysis using the maximum likelihood method with the mitochondrial cytochrome *COI* gene. *Ornithodoros sonrai* was employed as the outgroup. Black arrows show the sequences analyzed in this study. GenBank accession numbers for other sequences are presented with the sequence name. Branch numbers signify the bootstrap support levels (1000 replicates), and the scale bar shows the number of substitutions for each nucleotide.

In total, 576 ticks were collected in the study. Most ticks were partially fed. All of them, including 340 adults and 236 nymphs, were identified as *Haemaphysalis longicornis* by their morphological characteristics. Tick species were also molecularly identified using universal primers for the *COI* gene (expected size 710 bp) to avoid potential mistakes in the morphological identification. Both the morphological and molecular analyses identified all the ticks as *H. longicornis*. Furthermore, the nucleotide sequences from the representative ticks based on the developmental stage and collected region were assessed for the data analysis. The *COI* gene sequences obtained in this study shared close genetic relationships with *H. longicornis* (97.7–99.9% nucleotide identity). A phylogenetic tree was created according to the *COI* genes documented from several tick sequences deposited in GenBank (Figure 1).

### 3.2. Identification of TBPs

In total, 33.5% (193/576) of ticks, including 26.7% (63/236) of the nymphs and 38.2% (130/340) of the adults, were PCR-positive for at least one TBP (Table 1). TBPs were significantly more abundant (*p* = 0.0004) in the adult stage compared with the nymph stage. The 18S rRNA sequences of *Theileria orientalis* (29/576, 5.0%) were detected in the ticks. Among the positive samples, an additional genetic analysis revealed that the ticks were also positive for *T. orientalis* MPSP (29/576, 5.0%). The 16S rRNA sequences of *Anaplasma bovis* (13/576, 2.3%), *Anaplasma capra* (27/576, 4.7%), *Anaplasma phagocytophilum*-like *Anaplasma* spp. (APL) clades A (11/576, 1.9%) and B (3/576, 0.5%), and *Ehrlichia canis* (9/576, 1.6%) were detected in the ticks. Additionally, the 16S rRNA sequences of *Candidatus* Rickettsia longicornii (101/576, 17.5%) were discovered in the ticks. Among the positive samples, a supplemental genetic analysis revealed that the ticks were also positive for *Candidatus* R. longicornii *gltA* (101/576, 17.5%). *Candidatus* R. longicornii was the most abundant TBP in both developmental stages: 17.4% (41/236) of the nymphs and 17.6% (60/340) of the adults were PCR-positive. *A. bovis* (*p* = 0.0011), *A. capra* (*p* = 0.00001), and APL clade A (*p* = 0.0318) were significantly more abundant in the adult stage compared with the nymph stage. *A. bovis* was only identified in adults. TBPs were detected in varying proportions in different areas: in the nymph stage, 16.3% (13/80), 28.3% (17/60), and 34.4% (33/96) were detected in the northern, central, and southern areas, respectively, and in the adult stage, 16.9% (14/83), 35.1% (34/97), and 51.3% (82/160) were detected in the northern, central, and southern areas, respectively. *A. bovis* from the southern area (*p* = 0.0150) and *A. capra* from the central (*p* = 0.0444) and southern (*p* = 0.0039) areas were significantly more abundant in the adult stage compared with the nymph stage.

With regard to infection, 2.8% (16/576) of ticks represented multiple infections (Table 2). Ticks infected with more than one pathogen constituted 1.3% (3/236) of the nymphs and 3.8% of the adults (13/340). There were no significant differences in multiple infections between pathogens for each tick stage. However, multiple infections tended to be more abundant in the adult stage compared with the nymph stage. Among the infected ticks, 4.8% of the nymphs (3/63) and 10% (13/130) of the adults carried more than one pathogen species. Three (0.5%) were coinfected with *T. orientalis* and *Candidatus* R. longicornii, one (0.2%) was coinfected with *T. orientalis* and APL clade A, one (0.2%) was coinfected with *E. canis* and *A. capra*, three (0.5%) were coinfected with *Candidatus* R. longicornii and *A. bovis*, four (0.7%) were coinfected with *Candidatus* R. longicornii and *A. capra*, three (0.5%) were coinfected with *Candidatus* R. longicornii and APL clade A, and one (0.2%) was coinfected with *T. orientalis*, *A. bovis*, and *Candidatus* R. longicornii. *Bartonella* spp. and SFTSV were not detected in this study.

### 3.3. Molecular and Phylogenetic Analyses

**Figure 2:**
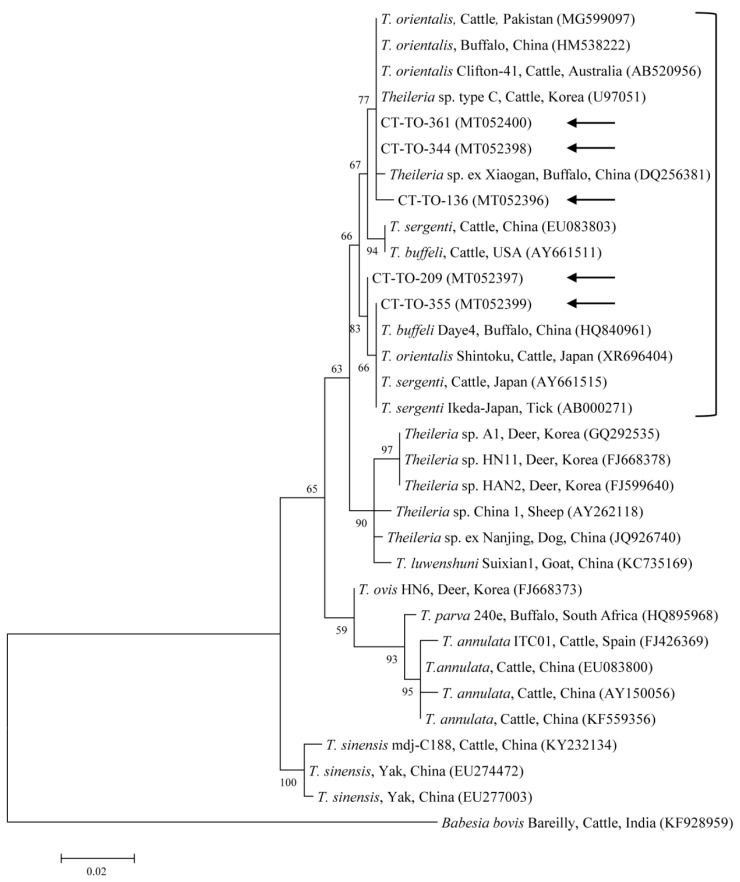
Phylogenetic tree of *Theileria* spp. based on sequences of the 18S rRNA gene. The tree was created using the maximum likelihood method. Black arrows show the sequences analyzed in the present study. *Babesia bovis* was employed as the outgroup. GenBank accession numbers of other sequences are presented with the sequence name. Branch numbers signify the bootstrap support levels (1000 replicates), and the scale bar shows the number of substitutions for each nucleotide.

**Figure 3:**
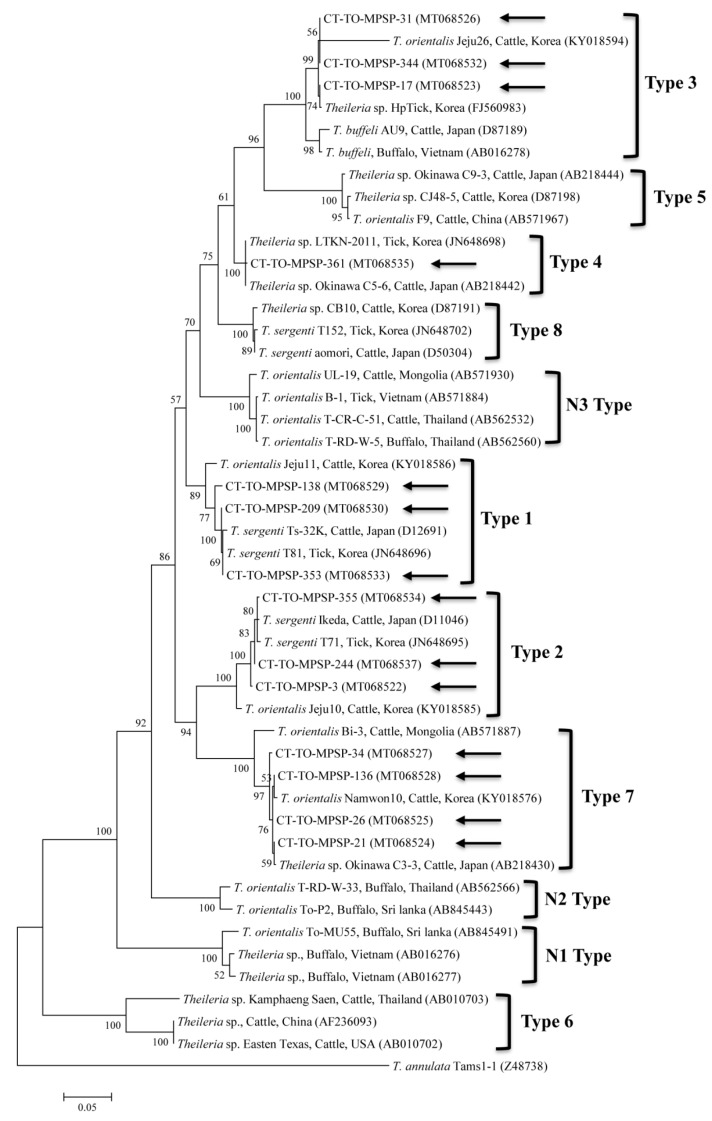
Phylogenetic tree of *Theileria* spp. based on sequences of the major piroplasm surface protein (MPSP) gene. The tree was created using the maximum likelihood method. Black arrows show the sequences analyzed in the present study. *Theileria annulata* was employed as the outgroup. GenBank accession numbers of other sequences are presented with the sequence name. Branch numbers signify the bootstrap support levels (1000 replicates), and the scale bar shows the number of substitutions for each nucleotide.

**Figure 4:**
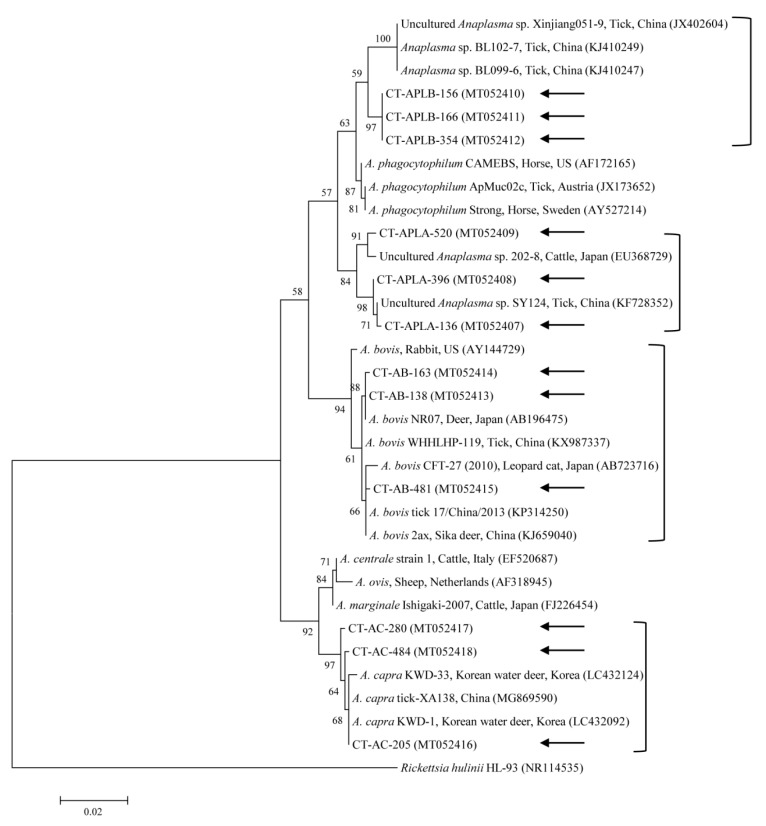
Phylogenetic tree of *Anaplasma* spp. based on sequences of the 16S rRNA gene. The tree was created using the maximum likelihood method. Black arrows show the sequences analyzed in the present study. *Rickettsia hulinii* was employed as the outgroup. GenBank accession numbers of other sequences are presented with the sequence name. Branch numbers signify the bootstrap support levels (1000 replicates), and the scale bar shows the number of substitutions for each nucleotide.

**Figure 5:**
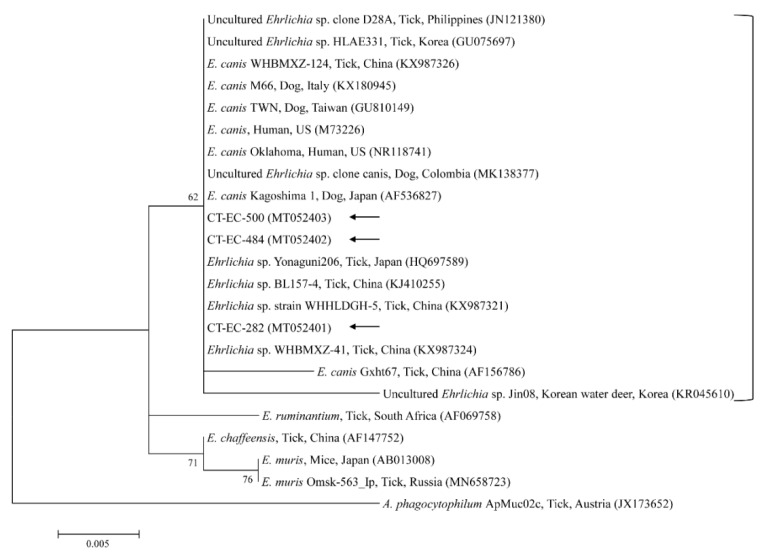
Phylogenetic tree of *Ehrlichia* spp. based on sequences of the 16S rRNA gene. The tree was created using the maximum likelihood method. Black arrows show the sequences analyzed in the present study. *Anaplasma phagocytophilum* was employed as the outgroup. GenBank accession numbers of other sequences are presented with the sequence name. Branch numbers signify the bootstrap support levels (1000 replicates), and the scale bar shows the number of substitutions for each nucleotide.

**Figure 6:**
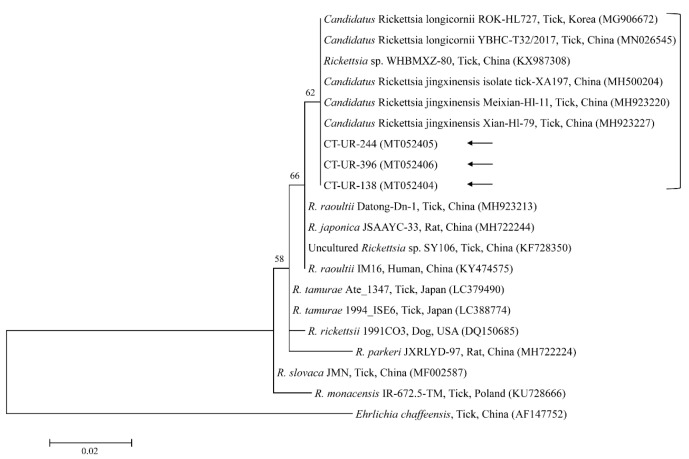
Phylogenetic tree of *Rickettsia* spp. based on sequences of the 16S rRNA gene. The tree was created using the maximum likelihood method. Black arrows show the sequences analyzed in the present study. *Ehrlichia chaffeensis* was employed as the outgroup. GenBank accession numbers of other sequences are presented with the sequence name. Branch numbers signify the bootstrap support levels (1000 replicates), and the scale bar shows the number of substitutions for each nucleotide.

**Figure 7:**
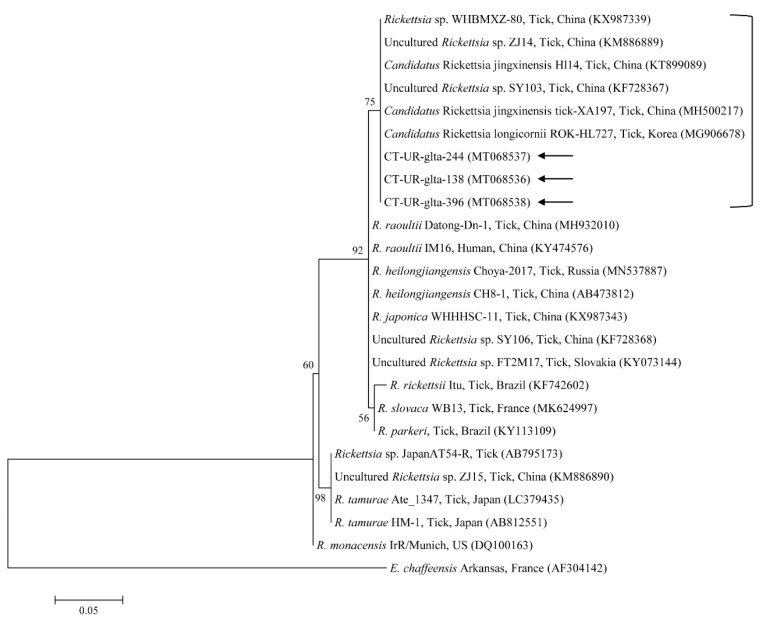
Phylogenetic tree of *Rickettsia* spp. based on sequences of the *gltA* gene. The tree was created using the maximum likelihood method. Black arrows show the sequences analyzed in the present study. *Ehrlichia chaffeensis* was employed as the outgroup. GenBank accession numbers of other sequences are presented with the sequence name. Branch numbers signify the bootstrap support levels (1000 replicates), and the scale bar shows the number of substitutions for each nucleotide.

Phylogenetic analyses showed that the 18S rRNA (Figure 2) and MPSP (Figure 3) nucleotide sequences of *Theileria* spp., 16S rRNA nucleotide sequences of *Anaplasma* spp. (Figure 4) and *E. canis* (Figure 5), and 16S rRNA (Figure 6) and *gltA* (Figure 7) nucleotide sequences of *Rickettsia* spp. were clustered with previously documented sequences.

The five sequences of *T. orientalis* found in the present study shared a 97.4–98.8% identity with the 18S rRNA sequence. They also shared a 97.6–99.7% identity with the 18S rRNA sequences in previously reported *T. orientalis* isolates. The *T. orientalis* MPSP gene sequences were classified into five genotypes: types 1, 2, 3, 4, and 7. Among the 29 sequences, 11, 10, 3, 1, and 4 isolates were assigned to types 1, 2, 3, 4, and 7, respectively. The three representative sequences of types 1 and 2 found in the present study shared a 98.7–99.8% and 98.8–99.3% identity with the MPSP sequence, respectively. They also shared a 99.5–99.8% and 99.3–99.5% identity with the MPSP sequences in previously reported *T. orientalis* isolates, respectively. The three, one, and four sequences of types 3, 4, and 7 that we found each shared a 99.6–100%, 100%, and 97.4–99.9% identity with the MPSP sequence, respectively. Each of them also shared a 98.1–99.8%, 98.3–99.8%, and 97.9–99.3% identity with the MPSP sequences in previously reported *T. orientalis* isolates, respectively.

The three representative sequences of *A. capra* herein shared a 99.7–99.8% identity with the 16S rRNA sequence. They also shared a 99.7–100% identity with the 16S rRNA sequences in previously reported *A. capra* isolates. We determined that the three representative sequences of APL clade A shared a 98.7–99.8% identity with the 16S rRNA sequences and a 98.7–99.8% identity with the 16S rRNA sequences in previously reported APL clade A isolates. Our three sequences of APL clade B shared a 100% and 98.6% identity with the 16S rRNA sequence and reported APL clade B isolates, respectively. Similarly, the three representative sequences of *A. bovis* shared a 99.4–99.9% identity with the 16S rRNA sequences and a 99.2–100% identity with other *A. bovis* isolates.

The three representative sequences of *E. canis* observed in our study shared a 100% identity with the 16S rRNA sequence and a 99.0–100% identity with previously reported *E. canis* isolates. Our three representative sequences of *Candidatus* R. longicornii shared a 100% identity with 16S rRNA and *gltA* sequences and a 100% and 98.9–99.2% identity with 16S rRNA and *gltA* sequences in previously reported isolates, respectively.

The representative sequences ascertained in this study were submitted to GenBank. The accession numbers are as follows: MT068539–MT068544 (*H. longicornis*), MT052396–MT052400 (*Theileria* spp. 18S rRNA), MT068522–MT068535 (*Theileria* spp. MPSP), MT052407–MT052418 (*Anaplasma* spp.), MT052401–MT052403 (*E. canis*), MT052404–MT052406 (*Rickettsia* spp. 16S rRNA), and MT068536–MT068538 (*Rickettsia* spp. *gltA*).

## 4. Discussion

In our study, only *H. longicornis*, including 340 adults and 236 nymphs, was found in cattle by both morphological and molecular methods. These findings are consistent with the result of a previous Korean study, which found *H. longicornis* (900/903, 99.7%) and *Ixodes* spp. (3/903, 0.3%) in cattle [10]. In another study [25], *H. longicornis* (15,020/19,821, 75.8%), *H. flava* (3889/19,821, 19.6%), and *I. nipponensis* (912/19,821, 4.6%) were identified from various habitats. *H. longicornis* is the most frequently identified species in Korea. The climate of Korea is steadily becoming subtropical due to global warming. The emergence of endemic TBPs might be associated with climate-driven changes to their geographic ecology and range. In the present study, TBPs were more prevalent in the southern area in the nymph and adult tick stages. This biogeoclimatic difference may clarify the observed differences in the prevalence of ticks and TBPs. These findings show that an additional geographical study is needed to fully understand the tick populations and clarify the distribution of TBPs in animals.

In the present study, the prevalence of TBPs was 1.4-times higher in the adults (38.2%) than in the nymphs (26.7%). This result is similar to that of a previous study [26] in which the overall infection rate was 2.7-times higher in adults compared with nymphs, most likely due to the transstadial accumulation in the mature ticks. In addition, the prevalence of multiple infections was higher in the adults than in the nymphs. In total, 1.3% of the collected *H. longicornis* nymphs and 3.8% of the adults were infected with more than one disease agent, constituting 4.8% and 10% of all the infected nymphs and adults, respectively. The *COI* sequences from the collected *H. longicornis* showed a 97.6–99.7% nucleotide identity with known *COI* sequences of *H. longicornis* (Figure 1). *H. longicornis* is typically collected from grasslands and herbaceous vegetation throughout Korea [25].

In this study, *T. orientalis, A. bovis*, *A. capra*, APL clades A and B, *E. canis*, and *Candidatus* R. longicornii were detected in cattle ticks by the molecular analysis. Theileriosis, one of the most important tick-borne hemoprotozoan diseases, can affect domestic animals, most frequently sheep and cattle in subtropical and tropical zones, and it causes great economic losses [27]. The members of the taxonomic group encompassing *T. buffeli, T. sergenti,* and *T. orientalis* are very similar, and the separate taxonomy of this group is controversial. Based on molecular studies, the three parasites are classified as one species, *T. orientalis* [28]. Recently in Korea, 18S rRNA genes were detected as *T. orientalis* in ticks (3.7%, 21/566 pools) from cattle [10] and *T. orientalis* in cattle (23.2%, 69/298) [29]. Here, 15 *H. longicornis* nymphs and 14 *H. longicornis* adults were positive for the *T. orientalis* 18S rRNA gene. The prevalence of *T. orientalis* in ticks herein (5.0%) was lower than that in a cattle study (23.2%) in Korea [29]. Based on the sequence analysis of the MPSP gene, *T. orientalis* consists of at least 11 different MPSP genotypes, including types 1–8 and N1–N3 [28]. Of the 11 MPSP genotypes, the Ikeda group consists of types 2 and 7, and the Chitose group consists of types 1, 3, 4, 5, 8, and N-3 [30]. Types N-1 and N-2 appear to have low sequence homology to each other [30]. Type 6 has been found in yaks and cattle, and it is classified as *T. sinensis* [31]. Recently in Korea, types 1, 2, 4, and 8 of the *Theileria* MPSP gene (2.7%, 15/556) were identified in ticks from grazing cattle [9], and types 1–3 and 7 (41.3%, 57/138) were identified in cattle [32]. In the present study, types 1–4 and 7 of the *Theileria* MPSP gene (5%, 29/576) were identified in cattle ticks. To our knowledge, this is the first study to report the presence of *Theileria* MPSP genotypes 3 and 7 in ticks in Korea. Of the five MPSP genotypes identified in this study, types 1 (37.9%, 11/29) and 2 (34.5%, 10/29) were the most commonly detected in ticks. In previous studies, type 2 in cattle [32] and types 2 and 4 in ticks from cattle [9] were also predominant. Types 1 (Ikeda) and 2 (Chitose) have been most commonly linked to clinical diseases. Of these, type 2 is more pathogenic [28], and it causes high parasitemia, severe anemia, and sometimes death. Therefore, it is imperative to conduct additional studies to identify the relationship between clinical signs and pathogenic types by determining the MPSP genotypes of *T. orientalis* that are related to animals and ticks.

The genus *Anaplasma* contains obligate intracellular Gram-negative bacteria belonging to the order Rickettsiales and the family Anaplasmataceae [33]. There are seven formally recognized species of *Anaplasma* (*A. phagocytophilum*, *A. centrale*, *A. marginale*, *A. platys, A. bovis*, *A. ovis*, and *A. caudatum*). *A. capra* represents a probable eighth species, and it has been argued as a new *Anaplasma* species whose name has not yet been formally recognized [34]. *A. capra* was recently identified from goats in China as a new tick-transmitted emerging zoonotic pathogen owing to its isolation from human blood (5.9%, 28/477) after tick bites [35]. It remains uncertain whether *A. capra* is pathogenic to both humans and animals, but if it is confirmed as such, it could pose a significant public health risk, the same as *A. phagocytophilum* [35]. In Korea, *A. capra* was formerly identified in cattle (0.4%, 5/1219) [12] and in Korean water deer (17.7%, 35/198) [36]. In the present study, one *H. longicornis* nymph and 26 *H. longicornis* adults tested positive for *A. capra*. The prevalence of *A. capra* in ticks herein (4.7%) was higher than that in a cattle study (0.4%) in Korea [12]. To our knowledge, this is the first study to report the presence of *A. capra* in ticks in Korea. *A. capra* is an emerging human pathogen in ticks parasitizing cattle and other animals in Korea. However, the vector capability of ticks for the transmission of *A. capra* is still unclear and needs additional evaluation for public health control. 

*A. phagocytophilum* is the causative pathogen of granulocytic anaplasmosis in various species, such as humans, dogs, goats, horses, sheep, and cattle [37]. The rapid and accurate diagnosis of pathogenic and zoonotic diseases, such as human granulocytic anaplasmosis, is required for the risk estimation in TBP control programs [38]. Therefore, it is meaningful to differentiate between pathogenic *A. phagocytophilum* and APL species that do not cause clinical signs in infected animals and that are presently considered non-pathogenic [38]. APL clade A was detected in cattle (2.6%, 20/764) in Korea [13]. Several APL clades have also been reported in other countries: APL clade A was detected in cattle (2.0%, 1/50) and ticks (2.4%, 2/85) in Japan [39], APL clade B was found in ticks (0.8%, 3/388 pools) in China [40], and APL clades A and B were detected in cattle (1.9%, 7/367; 0.5%, 2/367), sheep (7.0%, 25/355; 5.4%, 19/355), and goats (13.3%, 32/241; 5.0%, 12/241) in Tunisia [38]. In the present study, APL clade A was detected in one *H. longicornis* nymph and 10 *H. longicornis* adults, and one *H. longicornis* nymph and two *H. longicornis* adults were positive for APL clade B. The prevalence of APL clade A in ticks herein (1.9%) was lower than that in a cattle study (2.6%) in Korea [13]. To our knowledge, this is the first study to report the presence of both APL clades A and B in ticks from Korea. Additional studies are required to provide more information on the molecular background and to trace the evolutionary tree of novel *Anaplasma* species. *A. bovis,* a monocytotropic species, has been reported in ruminants in numerous countries [41]. *A. bovis* was formerly detected in Korea in cattle (1.0%, 12/1219) [12], in ticks (7.5%, 20/266 pools) from Korean water deer [11], in *H. longicornis* ticks (2.5%, 1/40 pools) from native Korean goats [14], and in *H. longicornis* ticks (1.0%, 5/506 pools) [8]. In the present study, 13 *H. longicornis* adults were positive for *A. bovis*. The prevalence of *A. bovis* in ticks herein (2.3%) was higher than that in a cattle study (1.0%) in Korea [12]. *A. bovis* was the only species of TBPs that was not found in the nymph stage. Meanwhile, in our previous study [14], *A. bovis* was only detected in the nymph stage of *H. longicornis* ticks from goats. In another study [42], *A. bovis* was detected in all tick stages, including larvae, nymphs, and adults. Thus, this appears to be an issue of the number of ticks tested. If we were to collect more ticks, we would be more likely to detect *A. bovis* in nymphs.

Canine monocytic ehrlichiosis is a systemic infection in dogs caused by *E. canis*, which is transmitted by *Rhipicephalus sanguineus* sensu lato, known as the brown dog tick [43]. *E. canis* was previously identified in Korea in *H. longicornis* and *Ixodes turdus* (1.1%, 18/1638 pools) and small mammals (12.0%, 51/424) [7], in *H. longicornis* ticks (1.2%, 6/506 pools) [8], and in *H. longicornis* from cattle (22.3%, 126/566 pools) [10]. Moreover, reports have revealed human infections of *E. canis* in Venezuela [44] and a novel genotype of *E. canis* in a human from Costa Rica [45]. The presence of *E. canis* in human samples is likely associated with the high prevalence of this pathogen in ticks and dogs [45]. In our study, four *H. longicornis* nymphs and five *H. longicornis* adults tested positive for *E. canis.* Continuous monitoring for infected ticks and reservoir hosts is required to ensure the health and safety of animals and the public against the risks of TBP exposure.

*Rickettsia* spp. are emerging or re-emerging pathogens with public health importance [46]. Obligate intracellular bacteria belong to the spotted fever group (SFG) and cause tick-borne rickettsioses [47]. In the present study, one SFG rickettsia with the *Candidatus* status was identified in the ticks. A potentially new SFG rickettsia classified into the putative novel subgroup “*Candidatus* R. longicornii” has been detected in Korea, including in *H. longicornis* ticks (43.2%, 79/183) [48], in *H. longicornis* ticks (16.7%, 52/311 pools) [49], and in *H. longicornis* ticks (45%, 18/40 pools) from native Korean goats [14]. These pathogens were clustered together in a subgroup that represented a sister taxon separate from the known subgroups of SFG rickettsiae. Gene fragment sequences reported in GenBank for rickettsial isolates from *H. longicornis* in Korea, Japan, and China have uncertain taxonomic statuses and unidentified pathogenicity that are most likely correlated to *Candidatus* R. longicornii or to a very closely associated species [48]. In addition, the XY118 (KU853023) isolate was detected from a patient, representing its possible pathogenicity in humans. Further studies are required to determine the pathogenicity of this novel pathogen. Here, *Candidatus* R. longicornii was detected in 41 *H. longicornis* nymphs and 60 *H. longicornis* adults. Additional research is necessary to identify other novel *Rickettsia* spp. in ticks and animals in Korea.

The bootstrap support levels need to be evaluated cautiously. As a limitation in this study, some bootstrap values were low. This could be due to using small amplification fragments of genes for the phylogenetic analysis. Therefore, further studies are needed to analyze longer fragments of genes in a phylogenetic analysis for a better presentation of the data.

## 5. Conclusions

In the present study, seven TBPs were detected in cattle *H. longicornis* ticks from Korea, including human pathogens of *A. capra*, *E. canis*, and *Candidatus* R. longicornii. Overall, 33.5% of ticks harbored at least one TBP. In 1.3% of the nymphs and 3.8% of the adults, we found more than one TBP. Among them, *Candidatus* R. longicornii was the most prevalent. To our knowledge, this is the first study to report the presence of the pathogens *T. orientalis* MPSP genotypes 3 and 7, *A. capra*, and APL in ticks from Korea. Cattle ticks may be maintenance hosts for many TBPs, and veterinary and medical clinicians should be aware of their high probability of infection and clinical complexity in humans. Our results show that ticks parasitizing cattle could be possible maintenance hosts for TBPs, and because of the zoonotic pathogenic importance of TBPs, we need to increase the awareness of their wide distribution and adopt measures to prevent their spread. Moreover, this study shows that coinfections are represented and thus should be considered in the diagnosis of TBPs, and that additional studies in Korea are needed in the future.

## Figures and Tables

**Table 1:** Prevalence of tick-borne pathogens (TBPs) detected in *H. longicornis* ticks parasitizing cattle in Korea, 2014–2018.

Region	Stage	No. of Ticks		No. Ticks Positive (%)							
			*T. orientalis* (18S)	*A. bovis* (16S)	*A. capra* (16S)	APL clade A (16S)	APL clade B (16S)	*E. canis* (16S)	*Candidatus* R. Longicornii (16S)	Total
Northern	Nymph	80	4 (5.0)	0	0	0	0	0	9 (11.3)	13 (16.3)
	Adult	83	3 (3.6)	0	3 (3.6)	0	0	0	8 (9.6)	14 (16.9)
Central	Nymph	60	5 (8.3)	0	0	0	0	1 (1.7)	11 (18.3)	17 (28.3)
	Adult	97	4 (4.1)	3 (3.1)	7 (7.2) *	4 (4.1)	0	1 (1.0)	15 (15.5)	34 (35.1)
Southern	Nymph	96	6 (6.3)	0	1 (1.0)	1 (1.0)	1 (1.0)	3 (3.1)	21 (21.9)	33 (34.4)
	Adult	160	7 (4.4)	10 (6.3) *	16 (10.0) *	6 (3.8)	2 (1.3)	4 (2.5)	37 (23.1)	82 (51.3) *
Subtotal	Nymph	236	15 (6.4)	0	1 (0.4)	1 (0.4)	1 (0.4)	4 (1.7)	41 (17.4)	63 (26.7)
	Adult	340	14 (4.1)	13 (3.8) *	26 (7.6) *	10 (2.9) *	2 (0.6)	5 (1.5)	60 (17.6)	130 (38.2) *
Total		576	29 (5.0)	13 (2.3)	27 (4.7)	11 (1.9)	3 (0.5)	9 (1.6)	101 (17.5)	193 (33.5)

* Designates significant differences in prevalence (*p* < 0.05) between the two different stages. *A*., *Anaplasma*; APL: *Anaplasma phagocytophilum*-like *Anaplasma* spp.; *E*., *Ehrlichia*; *R*., *Rickettsia*; *T., Theileria*; 16S, 16S rRNA; 18S, 18S rRNA.

**Table 2:** Multiple infections of TBPs detected in *H. longicornis* ticks parasitizing cattle in Korea, 2014–2018.

Region	Stage	No. of Ticks	No. Mixed Infections (%)							
			*T. orientalis/* *Candidatus* R. Longicornii	*T. orientalis*/ APL Clade A	*E. canis*/ *A. capra*	*Candidatus* R. Longicornii/ *A. bovis*	*Candidatus* R. Longicornii/ *A. capra*	*Candidatus* R. Longicornii/ APL Clade A	*T. orientalis/* *A. bovis/* *Candidatus* R. Longicornii	Total
Northern	Nymph	80	0	0	0	0	0	0	0	0
	Adult	83	0	0	0	0	0	0	0	0
Central	Nymph	60	0	0	0	0	0	0	0	0
	Adult	97	1 (1.0)	0	0	1 (1.0)	1 (1.0_	1 (1.0)	0	4 (4.1)
Southern	Nymph	96	1 (1.0)	0	0	0	1 (1.0)	1 (1.0)	0	3 (3.1)
	Adult	160	1 (0.6)	1 (0.6)	1 (0.6)	2 (1.3)	2 (1.3)	1 (0.6)	1 (0.6)	9 (5.6)
Subtotal	Nymph	236	1 (0.4)	0	0	0	1 (0.4)	1 (0.4)	0	3 (1.3)
	Adult	340	2 (0.6)	1 (0.3)	1 (0.3)	3 (0.9)	3 (0.9)	2 (0.6)	1 (0.3)	13 (3.8)
Total		576	3 (0.5)	1 (0.2)	1 (0.2)	3 (0.5)	4 (0.7)	3 (0.5)	1 (0.2)	16 (2.8)

A., Anaplasma; APL: Anaplasma phagocytophilum-like Anaplasma spp.; E., Ehrlichia; R., Rickettsia; T., Theileria.

## Supplementary Materials

The following are available online at https://www.mdpi.com/2076-2607/8/5/728/s1, Table S1: Primers used for the detection of tick-borne pathogens in ticks from cattle in the present study.
