# Supplementary material for: Genotypic Analysis of Piroplasms and Associated Pathogens from Ticks Infesting Cattle in Korea
Source: Microorganisms. 2020 May 13;8(5):728. doi: 10.3390/microorganisms8050728 (PMC7284522; doi:10.3390/microorganisms8050728)
Supplement: Supplementary file 1 [file microorganisms-08-00728-s001.pdf]

Supplementary Table S1. Primers used for the detection of tick-borne pathogens in ticks from cattle in the present study

Organism	Gene	Primer	Sequence 5′ to 3′	Size (bp)	Amplification condition	Reference
Invertebrate	Mitochondrial cytochrome c oxidase subunit I	LCO1490	GGTCAACAAATCATAAAGATATTGG	710	95°C/5 min; 35 cycles: 95°C/60 s, 40°C/60 s, 72°C/30 s; 72°C/10 min	[16]
		HC02198	TAAACTTCAGGGTGACCAAAAAATCA			
Anaplasma spp.	16S rRNA	*		429	95°C/5 min; 45 cycles: 95°C/30 s, 59°C/30 s, 72°C/30 s; 72°C/10 min	Commercial AccuPower® Rickettsiales 3-Plex PCR Kit (Bioneer)
Ehrlichia spp.	16S rRNA	*		340	95°C/5 min; 45 cycles: 95°C/30 s, 59°C/30 s, 72°C/30 s; 72°C/10 min	Commercial AccuPower® Rickettsiales 3-Plex PCR Kit (Bioneer)
Rickettsia spp.	16S rRNA	*		252	95°C/5 min; 45 cycles: 95°C/30 s, 59°C/30 s, 72°C/30 s; 72°C/10 min	Commercial AccuPower® Rickettsiales 3-Plex PCR Kit (Bioneer)
Rickettsia spp.	*glt*A	Rsfg877	GGGGGCCTGCTCACGGCGG	380	95°C/10 min; 35 cycles: 95°C/60 s, 51°C/60 s, 72°C/60 s; 72°C/10 min	[19]
		Rsfg1258	ATTGCAAAAAGTACAGTGAACA			
Bartonella spp.	ITS-1	QHVE-OF	TTCAGATGATGATCCCAAGC	736	94°C/10 min; 35 cycles: 94°C/60 s, 55°C/60 s, 72°C/120 s; 72°C/5 min	[20]
		QHVE-OR	AACATGTCTGAATATATCTTC			
		QHVE-IF	CCGGAGGGCTTGTAGCTCAG	484		

		QHVE-IR	CACAATTTCAATAGAAC			
Severe fever with thrombocytopenia syndrome virus	S segment	NP-2F	CATCATTGTCTTTGCCCTGA	461	50°C/30 min; 95°C/15 min; 40 cycles: 95°C/20 s, 52°C/40 s, 72°C/30 s; 72°C/5 min	[21]
		NP-2R	AGAAGACAGAGTTCACAGCA			
		N2F	AAYAAGATCGTCAAGGCATCA	346	25 cycles: 94°C/20 s, 55°C/40 s, 72°C/30 s	
		N2R	TAGTCTTGGTGAAGGCATCTT			
Babesia spp. and *Theileria* spp.	18S rRNA	*		676	95°C/5 min; 35 cycles: 95°C/30 s, 59°C/30 s, 72°C/30 s; 72°C/5 min	Commercial AccuPower® Babesia & Theileria PCR Kit (Bioneer)
Babesia spp. and *Theileria* spp.	18S rRNA	BT-F1	GGTTGATCCTGCCAGTAGT	1077	94°C/10 min; 30 cycles: 95°C/30 s, 65°C/30 s, 72°C/45 s; 72°C/10 min	[17]
		BT-R2	TTGCGACCATACTCCCCCCA			
Anaplasma spp.	16S rRNA	EE1	TCCTGGCTCAGAACGAACGCTGGCGGC	1433	94°C/5 min; 35 cycles: 94°C/30 s, 50°C/30 s, 72°C/60 s; 72°C/10 min	[12, 13]
		EE2	AGTCACTGACCCAACCTTAAATGGCTG			
		EE3	GTCGAACGGATTATTCTTTATAGCTTGC	928	94°C/5 min; 35 cycles: 94°C/30 s, 50°C/30 s, 72°C/60 s; 72°C/10 min	
		EE4	CCCTTCCGTTAAGAAGGATCTAATCTCC			

* Commercial PCR kits were used for the detection of these pathogens.
